# A Peculiar Case of Darier Disease in Blaschkoid Distribution

**DOI:** 10.5826/dpc.1004a78

**Published:** 2020-10-26

**Authors:** Francesca Peccerillo, Sabrina Longhitano, Barbara Ferrari, Laura Bigi, Giovanni Pellacani, Giulia Odorici

**Affiliations:** 1 Department of Surgical, Medical, Dental and Morphological Sciences, Transplant, Oncological and Regenerative Medicine; Dermatology Unit, University of Modena and Reggio Emilia, Modena, Italy

**Keywords:** segmental Darier Disease, blaschkoid distribution, ATP2A2 mutation, dermoscopy, histopathology

## Introduction

Darier disease (DD) is an autosomal dominant genodermatosis belonging to the group of keratinization disorders that can affect epidermis, nails and mucous membranes, caused by a mutation in the ATP2A2 gene on chromosome 12q23-24.1, which has an important role in calcium signal transduction [1]. It is induced by haplo-insufficiency with variable expressivity, but with complete penetrance, in adults.

## Case Presentation

**Figure 1:**
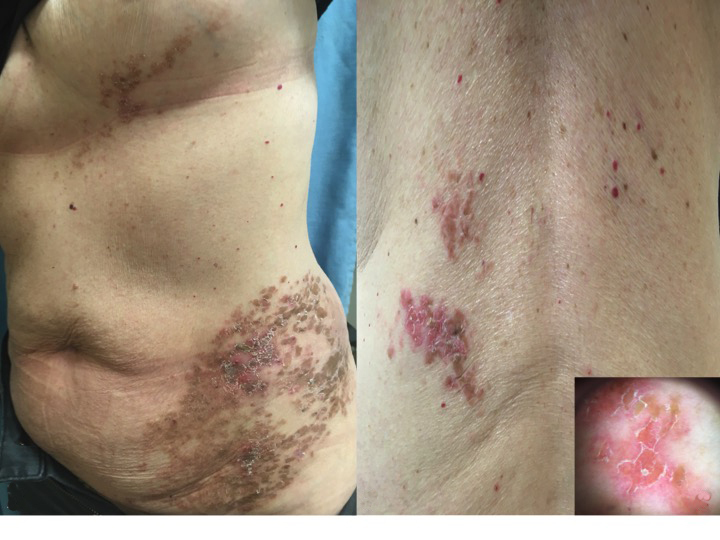
(A) Clinical examination revealed hyperkeratotic, reddish brown papules, segmental distributed along the left chest region. (B) Similar lesions were present on the back (lumbar region) following the Blaschko lines. (C) Dermoscopic examination showed polygonal-shaped brownish areas surrounded by a thin whitish halo (Canon G 16 digital camera; DermLite Foto, 3Gen).

We report a case of a 59-year-old woman with itching red-brown hyperkeratotic papules and plaques distributed on her left chest and left lumbar region, in blaschkoid distribution. The skin surrounding the lesions was normal (Figure 1, A and B). Dermoscopic examination revealed polygonal-shaped yellowish/brownish areas of various sizes surrounded by a thin whitish halo (Figure 1C). The patient stated that these lesions presented in a chronic and relapsing course since adolescence (after puberty) and had treated her cutaneous disease with topical homeopathic therapies. She denied comorbidities and family history of dermatologic disease.

**Figure 2:**
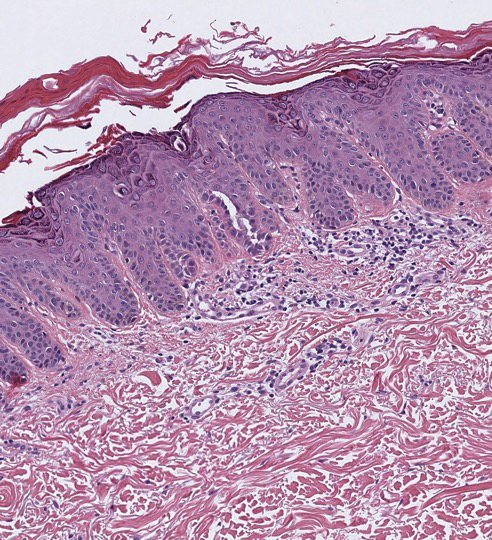
Histopathologic examination showed hyperkeratotic areas with acantholytic dyskeratosis in epidermis (corps ronds) and in the stratum corneum (grains) and formation of suprabasal cleft. The dermis showed sparse superficial perivascular lymphohistiocytic infiltrate. (H&E, ×20).

We performed a punch biopsy, and histopathological examination revealed acanthosis with acantholysis and dyskeratosis in the basal and suprabasal epidermal layers with formation of corp ronds and grains (Figure 2). Based on clinical and histopathological evaluations, a diagnosis of segmental DD was made.

## Conclusions

DD is characterized by specific dermatologic findings such as symmetrical keratotic papules on the seborrheic regions. Papules typically coalesce in plaques, usually localized in seborrheic areas of the trunk, scalp, forehead and flexures. Lesions may fluctuate in severity and are often exacerbated by ultraviolet light, heat, occlusion or stress [1].

Darier and White first described this disease independently in 1889; it is also known as keratosis follicularis. A localized manifestation of DD is a rare finding and was reported for the first time in 1906. This peculiar form is characterized by keratotic papules unilaterally distributed in streaks or whorls following Blaschko lines. It typically has a late onset and is aggravated by sunlight [1]. In this peculiar rare manifestation of DD, 2 phenotypes are recognized: type 1 is characterized by unilateral distribution along Blaschko lines, and type 2 presents widespread disease with localized areas of more severe involvement. Some authors estimate that these manifestations account for 10% of cases of DD with several variants: unilateral, linear, segmental or zosteriform. The localized forms may be clinically and histologically indistinguishable from nevi with acantholytic dyskeratosis. Differential diagnosis included the dermatological manifestation of clinical lesions following Blaschko lines, such as verrucous epidermal nevus, lichen striatus, and linear lichen planus. These peculiar clinical presentations reflect a de novo postzygotic somatic mutation in the heterozygous state [2]. In fact, over 100 different mutations have been identified in the classic form, and several of them have also been demonstrated in affected areas of mosaic DD patients [2]. In patients with unilateral localized disease, the distal nail manifestations of DD are often absent.

Our patient represents a peculiar case of segmental type 1 manifestation of DD. Although the treatment consisted predominantly of topical therapy, the patient refused any kind of topical application because of her faith in homeopathy and the relapsing course of her disease.
